# Phellodendri Cortex: A Phytochemical, Pharmacological, and Pharmacokinetic Review

**DOI:** 10.1155/2019/7621929

**Published:** 2019-04-01

**Authors:** Yue Sun, George Binh Lenon, Angela Wei Hong Yang

**Affiliations:** School of Health and Biomedical Sciences, RMIT University, Victoria, Australia

## Abstract

**Background:**

* Phellodendri Cortex* (PC) or Huang Bai. According to the scientific database of China Plant Species and Chinese pharmacopeia 2015 edition, PC has two main species which are* Phellodendron amurense Rupr *(PAR) or “Guan Huang bai” in Chinese and* Phellodendron chinense Schneid *(PCS) or “Chuan Huang bai” in Chinese. The crude drugs of PAR and PCS are also called* Phellodendri amurensis cortex* (PAC) and* Phellodendri chinense cortex* (PCC), respectively. The medicinal part of the plant is the dried trunk bark. PC has comprehensive therapeutic effects which include anti-inflammatory, antimicrobial, anticancer, hypotensive, antiarrhythmic, antioxidant, and antipyretic agents. The exact ingredients in PC and its species are not fully summarised.

**Aim of the Study:**

This study was designed to review and evaluate the pharmacological actions of compounds and to explore the pharmacokinetic knowledge of PC and its species and to also identify the chemical compound(s) with a potential therapeutic effect on atopic dermatitis.

**Methods:**

“Huang Bai” and its English, botanical, and pharmaceutical names were used as keywords to perform database search in Encyclopaedia of traditional Chinese Medicines, PubMed, EMBASE, MEDLINE, Science Direct, Scopus, Web of Science, and China Network Knowledge Infrastructure. The data selection criteria included all the studies that were related to the phytochemical, pharmacological, and pharmacokinetic perspectives of PC and its species or their active constituents. More importantly, the voucher number has been provided to ensure the genuine bark of PC used as the medicinal part in the studies.

**Results:**

140 compounds were summarized from PC and its species: specifically, 18 compounds from PCC, 44 compounds from PCS, 34 compounds from PAC, and 84 compounds from PAR. Obacunone and obaculactone are probably responsible for antiatopic dermatitis effect. PC and its species possess a broad spectrum of pharmacological actions including anti-inflammatory effect, antibacterial effect, antiviral effect, antitumor effect, antigout effect, antiulcer effect, neuroprotective effect, and antiatopic dermatitis effect. PC could widely distribute in plasma, liver, spleen, kidney, and brain. Berberine may be responsible for the toxic effect on the susceptible users with hemolytic disease or in the peripartum and neonatal period.

**Conclusions:**

The compounds of the crude bark of PC and its subspecies have showcased a wide range of pharmacological effects. Pharmacological efficacies of PC are supported by its diverse class of alkaloid, limonoid, phenolic acid, quinic acid, lignan, and flavonoid. Obacunone and obaculactone could be the bioactive compounds for atopic dermatitis management. PC and its subspecies are generally safe to use but extra care is required for certain conditions and group of people.

## 1. Introduction

*Phellodendri Cortex* (PC) is also known as “Huang bai” in Chinese and “Obaku” in Japanese. PC is a plant grown in China, Korea, Japan, Vietnam, and Far East of Russia. The earliest record of this plant was on “Shennong's Classic of Materia Medica” [1]. According to the scientific database of China Plant Species and Chinese pharmacopeia 2015 edition, PC has two main species which are* Phellodendron amurense Rupr* (PAR) or “Guan Huang bai” in Chinese and* Phellodendron chinense Schneid* (PCS) or “Chuan Huang bai” in Chinese. The crude drugs of PAR and PCS are called* Phellodendri amurensis cortex* (PAC) and* Phellodendri chinense cortex* (PCC), respectively. PAR and PCS are naturally grown in the Northeast and Southwest part of China, respectively [2]. According to the latest information from “information system of Chinese rare and endangered plants,” PAR is categorized as one of the second degrees of endangered plants. PAR and PCS could be interchangeably used in clinical application because both species contain similar chemical constituents [3]. According to the scientific database of China Plant Species and Chinese pharmacopeia 2015 edition, PC is categorized in the family of* Phellodendron Rupr*. The medicinal part of the plant is the dried trunk bark. PC had been viewed as one of the 50 fundamental herbs in Chinese herbalism. Traditionally, its medicinal part could exert therapeutic effects in various diseases such as meningitis, cirrhosis, dysentery, pneumonia, tuberculosis, etc. [4, 5]. Nowadays, PC has comprehensive therapeutic effects which include immune modulation, anti-inflammatory, antimicrobial, antibacterial, anticancer, hypotensive, antiarrhythmic, antioxidant, antigastric ulcer, and antipyretic agents, etc. [5]. According to the Clinical Chinese Materia Medica, 2006 edition, PC has a bitter flavor and cold nature and can enter the meridian of kidney, bladder, and large intestine. PC could clear heat, dry dampness, drain fire, eliminate steam, resolve toxin, and treat sores [6]. PC and its species contain various chemical derivatives. One of the important ones is alkaloids which contain berberine and jatrorrhizine. Both compounds were proven to be effective against some type of tumours, infections, neurological diseases [7]. To further acquire the knowledge on PC, a systematic review of its phytochemical, pharmacological, and pharmacokinetic properties is required. The aim of this study is to review and evaluate the pharmacological actions of compounds as well as to explore the pharmacokinetic knowledge of the PC and its species. The chemical compounds that exert therapeutic effect for atopic dermatitis are also desired.

## 2. Methods

Data were searched from the following databases until May 2018: Encyclopaedia of Traditional Chinese Medicines; PubMed; EMBASE; MEDLINE; ScienceDirect; SCOPUS; Web of Science; China Network Knowledge Infrastructure. The keywords used for the literature search included: Huang Bai and its English, botanical, and pharmaceutical names. The selection criteria included process controls of the herbal substances, reporting reference standards such as authentication of reference materials and profile chromatograms, and analytical procedures and validation data. Papers in English or Chinese language are included for this review. Scientific rigidity was determined by the chemical markers of herbs through the use of strict parameters in testing, quantitative, and qualitative measures of the bioactive components, such as high-performance liquid chromatography, fingerprint spectrum, correlations differentiation, and stability evaluation, reference standards, and toxicological assessments. Plant voucher specimens are a guarantee for traceability of the plant material and data verification for other researchers or commercial purposes [60]. The chemical formulas of the compounds of PC and its species were acquired from selected studies. Chemical structures and molecular weights were extracted from* ChemDraw professional 170*.

## 3. Results

**Figure 1:**
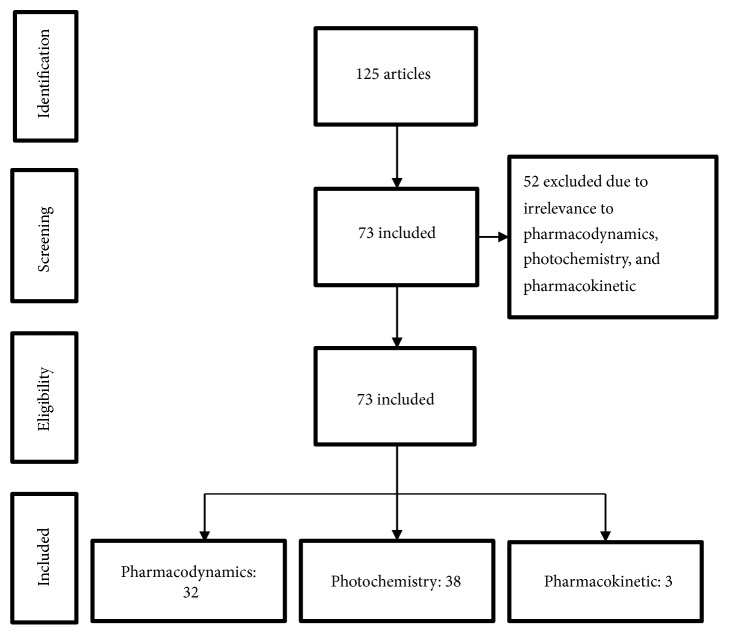
PC study selection flowchart.

A total of 125 papers were identified through the literature search. Fifty-two papers were excluded based on the reasons for nonpharmacodynamic, nonphytochemistry, and nonpharmacological studies. 73 studies fitted the selection criteria. Among these articles, 32 studies are about pharmacodynamics, 38 studies are about phytochemistry, and 3 studies are about pharmacokinetics (Figure 1).

## 4. Bioactive Compounds

The crude barks of PC and its species contain alkaloids, isoquinoline alkaloid, limonoids, phenolic acid, quinic acid, lignans, and flavonoid, and so on. A summary of the bioactive compounds of the PC and its species reported in the included studies is presented in Table 1. Molecular formula, molecular weight, and chemical structure of the major constituted alkaloids of PC, including berberine, palmatine, and jatrorrhizine, are listed in Tables 2, 3, 4, and 5. They could exert a broad spectrum of pharmacological influence which contains antimicrobial, anti-inflammation, antitumor, antidepressant, and antiulcer effects [47]. Limonoids including limonin and obakunone play an important role in the anti-inflammatory effects of PC [11]. Phenolic acid or phenol carboxylic acids belong to aromatic acid compound substances which are characterized by a phenolic ring and an organic carboxylic acid function [61]. According to the description on Buchler quinine plant in Braunschweig, Germany, quinic acid is a natural sugary compound which can be found in multiple plants such as well-known coffee beans and tobacco leaves. Based on the description on PubChem, lignans affiliate a class of dibenzylbutane derivatives which exists in plants and in body fluids such as bile, serum, urine, etc. These compounds have anticancer potential. Quercetin belongs to flavonoids which can reduce coronary heart disease according to PubChem data. Two species of PC, PCS and PAR, can be differentiated based on the contents of the chemical compounds. Specifically, the 2005 edition of Chinese Medicinal encyclopedia described the PCS's minimal content of berberine hydrochloride and phellodendron hydrochloride to be 3.0% and 0.34%, respectively; the PAR's minimal content of berberine hydrochloride and palmatine hydrochloride is 0.60% and 0.30%, respectively [62].

## 5. Pharmacology

### 5.1. Anti-Inflammatory Effects

The PAR extract could efficiently adjust lipopolysaccharide (LPS)-induced release of nitric oxide (NO) and inducible nitric oxide sythase (iNOS) production in microglia of both BV2 cells and mice. Besides, PAR extract could also attenuate the LPS-stimulated release of tumor necrosis factor-*α* (TNF-*α*) and interleukin 1*β* (IL-1*β*) from microglia. More importantly, the latter mechanism is more significant than the previous one in terms of IL-1*β* release. The inhibition of NO suggested that the extract of PAR probably could affect NO-induced neuronal cell death [28]. Another study had also vindicated the anti-inflammatory effect of PC extract on ear swelling model of mice. Magnoflorine and phellodenrine belong to alkaloids isolated from PC that are the effective compounds against oxazolone-induced contact-delayed type hypersensitivity (DTH) reaction induced by picryl chloride. Another mechanism in this study is that PC could reduce myeloperoxidase (MPO) activity to its utmost by restraining leukocyte mobility and/or a secretory activity by phellodendron. In contrast, PC extract exerts no effect on phospholipase A2 (PLA_2_) activity and less effect in arachidonic acid (AA) induced swelling [29]. PC showcased the inhibitory effect on the build-up of NO in LPS-stimulated macrophage Raw 264.7 cells and inhibited iNOS expression. In contrast, PC has no effect on cyclooxygenase-2 (COX-2) expression in LPS-induced RAW 264.7 cells [30]. PCC and PAC could not only shrink the size of edema, but also reduce the activity of MPO and the content of reactive oxygen species (ROS) caused by 12-O-tetradecanoyl-phorbol-13-acetate (TPA). They can also restrain the levels of TNF-*α*, Il-1*β*, IL-6, and COX-2 in mice treated by TPA. Remarkably, there are a number of chemical compounds in these two species of PC including berberine, palmatine, and phellodendrine. And they are viewed as the anti-inflammatory active candidates. In addition, they may jointly take effect in this regard [3]. The nonalkaloid PAR extract suppressed NO production; besides, limonin and obakunone significantly downregulated NO production and iNOS gene expression via an nuclear factor-*κ*B (NF-*κ*B)-mediated pathway [11]. PC could reverse the airway inflammation by reducing the infiltration of inflammatory cells and releasing of inflammatory mediators into the affected lung and airways. This could vindicate its applications on the infectious pulmonary diseases [31].

### 5.2. Antimicrobic Effects

For the antibacterial effect, the study showed both aqueous and ethanol extracts of PAR exerted an intermediate antimicrobial effect. Besides, PAR extracts had a slightly better effect on gram-positive bacteria than gram-negative one because of different sensitivity. The most sensitive bacteria is Streptococcus pyogenes [33]. For the bacteria in the oral cavity, PC could have a strong inhibitory effect on Porphyromonas gingivalis; moderate inhibitory effect on Streptococcus mutans; partial effect on Streptococcus sanguis; no effect on Streptococcus mitis [34]. Propionibacterium acnes which are the culprit for acne is active to PC which is one of the crude herbs in the clinical trial and the second best herb in terms of antiacne activity in this study [39]; Mycoplasma hominis which causes infections on humans genital tracts and respiratory tracts is susceptive to PC, and the susceptibility rate is 93% [35]. Salmonellosis which is responsible for food poisoning is vulnerable to PC extract because it can lower the IgG levels and induce TNF-*α* expression in RAW264.7 cells [36]. In case of PAR, berberine could restrain the bacterial adhesion of Staphylococcus aureus and intracellular invasion into human gingival fibroblasts [37]. Moreover, berberine could attenuate the aminoglycoside resistance of P. aeruginosa, A. xylosoxidans, and B. cepacia in the MexXY-dependent manner. It also inhibits MexXY- or MexVW-mediated resistance of P. aeruginosa mutants, synergistically restrains MexXY-mediated gentamicin resistance in P. aeruginosa mutants, and enhances the synergistic effect of piperacillin and amikacin in multimedication resistant P. aeruginosa strains. The extract of PCS significantly downregulated minimal inhibitory concentrations (MIC_s_) of amikacin and gentamicin in the two multimedication resistant P. aeruginosa strains [38]. PC showed its potential on the inhibition of Propionibacterium acnes strains. Its MIC_50_ and MIC_90_ were 24 *μ*g/ml and 190 *μ*g/ml, respectively [39]. For fungal infections, the monomers of PC showed antifungal activity through compromising the integrity of fungal cell wall and cell membrane and increasing the expressions of energy metabolic genes. Therefore the life expectancy of Microsporum Canis is shortened. Furthermore, the mingling use of palmatine hydrochloride and berberine hydrochloride could effectively treat Microsporum Canis induced dermatomycosis in rabbit [40]. For virus infections, the ethanol extract of PAR exerted moderate effect against Herpes Simplex Virus by either interrupting virion envelope structures or disguising as indispensable viral compounds for absorption or infiltration of host cells [33]. Another study had proved that PAR has a broad spectrum of functions against virus-like VSV-GFP, PR8-GFP, NDV-GFP, HSV-GSP, H3-GFP, and EV-71 in vitro and also has effects on different strains of influenza A such as H1N1, H5N2, H7N3, and H9N2 in vivo mice model [41].

### 5.3. Antitumor Effects

There are 3 compounds in PCS that have been found to resist three types of tumours which included leukemic cell lines K562 and HEL, breast cancer cell line MDA, and prostate cancer cell line PC3. The compound* [(21S, 23R) epoxy-24-hydroxy-21β, 25-diethoxy] tirucalla-7-en-3-one* has a relatively strong effect as Adriamycin against four tumors with the measurement of IC_50_;* toonaciliatin K* and* piscidinol A* have a comparably moderate effect in this regard [42]. There are 9 most effective compounds of PC for prostate cancer, namely,* magnoflorine-O-glucuronide, (p-hydroxybenzyl)-6, 7-dihydroxy-N-methyl tetrahydro iso-quinoline-7-O-p-D-glucopyranosid, magnoflorine, menisperine-O-glucuronide, menisperine, berberine, Jatrorrhizine, obaculactone*, and* obacunone* [43]. Polysaccharides from an aqueous extract of PCS act on cell-mediated stimulation and humoral immunity instead of tumor cell inhibition to exert tumoricidal activity. Specifically, some polysaccharides stimulate macrophages and NK cells via *β*-glucan-binding lectin site of complement receptor type 3 [44].

### 5.4. Antigout Effects

Si-Miao-Wan (SMW) formula had been proved to be effective for gout and gouty arthritis. In this formula, PC is the monarch herb which is the core ingredient to guide the other three herbs indicating that the alkaloids and organic acids in PC are the potential compounds for SMW. Alkaloids include candicine, oblongine, phellodendrine, tembetarine, magnoflorine, lotusine, n-methylterahydrocolumbamine, menisperine, noroxyhydrastinine, demethyleneberberine, tetrahydropalmatine, oxyberberine, armepavine, oxypalmatine, columbamine, jatrorrhizine, thalifendine, berberrubine, n-methyl canadine, palmatine, berberine, obaculactone, obacunone, and amurenlaetone B, and organic acids include neochlorogenic acid, chlorogenic acid, cryptogenic acid, cryptochlorogenin acid, caffeoyl-CH_2_-O-quinic acid, 3-O-feruloylqinic acid, ferulic acid, and sanleng acid [45]. Er-Miao-Wan formula is a modified version of SMW, which had been elucidated for its chief ingredient PCS which exerts potent hypouricemic effect [46].

### 5.5. Antiulcer Effects

Peptic ulcers are associated with psychological stress and mental illness. The middle dosage of PC extract could significantly reduce the levels of serotonin in the brain and noradrenaline in the adrenal gland. Both serotonin and noradrenaline take effect in the mental depression. Besides, for the molecule in PC, berberine has the ability to inhibit monoamine oxidase-A and modulate the brain noradrenaline, serotonin, and dopamine levels [47]. Another study revealed that PC could protect the gastric mucosa by reinforcing the gastric mucosal barrier through endogenous sulfhydryl compounds and diethyldithiocarbamate-sensitive compounds [48].

### 5.6. Antioxidant Effects

The antioxidant activity of PAR is proportional to its extract's concentration. In another aspect, the ethanol extract exhibited a better antioxidant effect because of its high concentration of phenolics and flavonoids than aqueous extract [33]. Phellodendrine from PC could play an antioxidant role by modulating the AKT/NF-*κ*B pathway in the zebrafish embryo. Besides, phellodendrine could undo the expression of AKT and NF-*κ*B, IKK, and COX-2 [49].

### 5.7. Sun Screening Effects

The sun screening effects of PC have been demonstrated through an experiment which was designed for sun screening effect of 50% alcohol extracts of 100 Chinese herbal medicines. The study showed PC could absorb 91.8% of ultraviolet-C, 79.1% of ultraviolet-B, and 50.7% of ultraviolet-A. It is regarded as highly effective for sun screening if the absorption rate of ultraviolet is higher than 90%. Therefore, PC could be a strong candidate for sunproof of ultraviolet-C [50]. Also, PC could also improve skin oxidative lesion induced by ultraviolet radiation via decreasing lipid peroxidation and increasing antioxidant enzymes activities [51].

### 5.8. Other Effects

PC stimulates longitudinal bone growth and chondrocyte proliferation via upregulating bone morphogenetic protein-2 (BMP-2) and insulin-like growth factor (IGF-1) expression in the growth cartilage [52]. Besides, PC could activate the fibrinogen system to take the hemostatic effect. This validated charcoaled PC could stop bleeding [53]. The extract of PC also shows neuroprotective effect through adjusting the PC-12 cell apoptosis which was induced by 1-methyl-4-phenylpyridinium (MPP^+^) and hindered the release of cytochrome C into the cytosol [54]. PAR could ease the symptoms of atopic dermatitis by decreasing the numbers of mast cells, serum levels of TNF-*α*, and INF- *γ* and the expression levels of cytokines [56]. PAR could delay or even prevent the progression of diabetic nephropathy by correcting the high blood sugar state, antioxidant enzyme system, and kidney malfunction and reversing histopathological changes inflicted by diabetes on kidney [57]. To further elaborate its mechanism on the compound level, berberine could attenuate the renal malfunction by inhibiting renal aldose reductase and decreasing oxidative stress [58]. Magnoflorine and phellodendrine could inhibit the immune response by suppressing local graft versus host reaction and induction phase of picryl chloride-induced delayed-type hypersensitivity [59]. To counter asthma attack, the extract of PCS inhibits tracheal smooth muscle contraction induced by high K^+^. Meanwhile, it could also block tracheal smooth muscle concentration induced by Nifedipine [6]. The pharmacological activities of PC and its related derivatives have been listed in Table 6.

## 6. Pharmacokinetics

According to Chinese Pharmacopeia (Edition 2015), phellodendrine has been used as one of the evaluating indexes of PC. Phellodendrine could be rapidly absorbed in tissues such as plasma, liver, spleen, kidney, and brain. Besides, kidney is the major distribution tissue and the target organ of phellodendrine. Furthermore, the experimental study on animal suggested that this constituent has no long-term build-up effect on the tissues. Intriguingly, the extract of phellodendrine could be found in the animal brain tissue indicating that this constituent may penetrate the common medication's biggest hurdle: blood-brain barrier. The maximum concentration time is at 5 minutes after intravenous administration. The elimination half-life is no longer than 2 hours [63]. Another constituent from PC is magnoflorine which shows low bioavailability and high absorption and elimination rates after oral and intravenous administration of this constituent in pure compound form. While its bioactivity can be dramatically increased, absorption and elimination rates can be significantly decreased after oral administration of the PC decoction. Similarly, with oral administration of mixture, magnoflorine (40 mg/kg) and berberine (696.4 mg/kg, the equivalent dosage in PC decoction), the bioavailability and absorption and elimination rates have a similar trend. It suggested berberine plays an important role in the drug-drug interaction with magnoflorine in the PC decoction. On the other hand, these findings also warn us that the mingling use of berberine and magnoflorine possibly increases the risk of toxicity which possibly gives some support to the theory that herbal medicine may achieve better therapeutic effects and fewer side effects [64]. When it comes to the different type of processed PC, they have different kinds of effects. For the raw PC, it could downregulate CYP1A2 and activate CYP3A4. As for rice-wine and salt-water processed PC, they can alter the activities of cytochrome P450. Also, rice-wine processed PC alone can counter the inhibitory effect of CYP1A2 and promote the induction of CYP3A4 [65].

### 6.1. Toxicity and Contraindication

Several studies have been conducted regarding the toxicology of PC applications. So far, no conclusive result has been reached due to the controversial results from different studies. The common allegation for PC application is neonatal jaundice and kernicterus. Due to this concern, it even causes the complete ban on the use of related herbs including PC in Singapore since 1978. Besides, according to the latest Singaporean official regulations for health supplements guideline, PC is still on the list of prohibited or restricted ingredients. However, according to a cohort study, under the guidance of Chinese medicine practitioners, the application of PC's berberine is clinically safe even in patients who have hematological diseases with profound cytopenia and multiple comorbidities. Despite these, some precautionary measures such as bilirubin and hemoglobin monitoring are still required for the patients who have underlining hemolytic disease. On the other hand, the restriction for PC is necessary for the users in their peripartum and neonatal period due to the concern of its aggravation risk for neonatal jaundice and kernicterus [66].

### 6.2. Processing, Differentiation, and Authentication

Traditional Chinese medicine (TCM) processing or preparation or “Pao Zhi” in Chinese is a unique technique and process in TCM. Pao Zhi is a technique to turn the raw herbs into decoction pieces. This technique must be performed under the guidance of TCM's theory to satisfy the different requirements of medicinal materials and special production processes. The quality of Pao Zhi directly affects the therapeutic effects of herbal medicine [67]. It has a time-honored history because as early as 5 B.C. during the Jin dynasty, “Leigong Treatise on the Preparation” was composed as a book for Pao Zhi. It systematically summarized the herbal processing techniques until the Jin dynasty. Intriguingly, it stated that the right way of using the bark of PC is to remove the coarse bark [68]. In the Song dynasty, Pao Zhi had regulated a mandatory process for Chinese medicinal product [67].

In terms of PC's processing, TCM doctors in history emphasized the importance of PC's processing and recorded 16 kinds of methods for PC's processing in the books. Nowadays, the most common types of processing are raw PC, PC fried with salt, PC fried with wine, PC fried with honey, and fried-to-charcoal PC [69]. However, it still lacks official standard when it comes to quantity and quality of the processed PC and its subspecies; some of the existing pioneer studies could explain this ambiguous and abstract concept in a scientific way. Besides, for PC's quality control, the most practical approach is to use thin layer chromatography (TLC) for qualitative and high-performance liquid chromatography (HPLC) for quantitative measurement [70]. According to the results of thin layer chromatography, water percentage in the raw PC is less than 10.0%, PC fried with salt is less than 8.0%, PC fried with wine is less than 7.0%, and PC fried with honey is less than 8.0%. Total ash content in the raw PC is less than 8.0%; acid insoluble ash content is less than 0.8%; PC fried with salt is less than 8.0%; acid insoluble ash content is less than 0.6%; PC fried with wine is less than 8.0%; acid insoluble ash content is less than 0.8%; PC fried with honey is less than 8.0%; acid insoluble ash content is less than 0.4%. For alcohol-soluble extract, the raw PC is more than 16.0%, PC fried with salt is more than 20.0%, PC fried with wine is more than 16.0%, and PC fried with honey is more than 22.0%. For percentage of phellodendrine, the raw PC is more than 0.41%, and berberine is more than 3.92%; PC fried with salt is more than 0.36%, and berberine is more than 3.89%; PC fried with honey is more than 0.37%, and berberine is more than 3.90% [69].

Previously, the differentiation of PCS and PAR is based on the experiences of the herbal professionals to distinguish the minor differences from their appearances in naked eyes and microscope. Due to the increasing mixing applications and counterfeits, more efficient and accurate approaches are required. It is noticeable that the mass fractions of obaculactone and obacunone have a plunging order among raw PC, PC fried with wine, and PC fried with salt. Therefore, it is reasonable to deduce limonin and obacunone have been undergoing a series of chemical reactions during herbal processing. For differentiation of PCS and PAR, the mass fractions of limonin and obacunone have a significant difference between PCS and PAR. The mass fraction of obaculactone and obacunone in PAR is 10 times higher than in PCS. As a result, limonin and obacunone could be utilized as the differential targeted chemical constituents for PC's differentiation [71]. HPLC method demonstrates that PC and charcoaled PC have a significant disparity in terms of characteristic chromatograms and chemical constituents. The peak numbers and proportions of characteristic chromatograms are reducing with the increasing temperature of charcoaled PC. On the other hand, due to the heating effect, berberine hydrochloride will transfer into berberrubine by losing one methyl. As a result, berberrubine is vindicated to be another targeted chemical constituent for charred PC identification [72]. For authentication of PC and its subspecies, berberine hydrochloride could be used as another targeted chemical constituent except for limonin and obacunone as mentioned above [73].

## 7. Discussion

This review work has illustrated the diverse bioactive properties of PC and its species associated with active pharmacological actions in vitro and in vivo. Its clinical applications which are demonstrated in these experimental studies indicated the potency of the bioactive compounds and its pharmacological effects.

The diverse derivatives are the backbone for the pharmaceutical efficacies of PC and its species. The alkaloids play a very significant role in this regard not only because they account for a great proportion of constituents in the whole herb, but also because these constituents are relatively well-studied compared to other constituents in other derivatives. Berberine, magnoflorine, palmatine, and phellodendrine are viewed as the anti-inflammatory active candidates in experimental studies. Besides, palmatine and berberine could exert antimicrobial effects. Berberine also has the ability to inhibit monoamine oxidase-A and modulate the brain noradrenaline, serotonin, and dopamine for antiulcer efficacy. The nonalkaloid in PAR extract suppressed NO production; besides, limonin and obakunone significantly downregulated NO production and iNOS gene expression via an NF-*κ*B-mediated pathway. [(21S, 23R) Epoxy-24-hydroxy-21*β*, 25-diethoxy] tirucalla-7-en-3-one, toonaciliatin K, and piscidinol have tumor-shrinking effects on four types of tumors. Polysaccharides from an aqueous extract of PCS act on cell-mediated stimulation and humoral immunity to exert tumoricidal activity.

Phellodendrine and magnoflorine have well absorption rate in the animal organs which could indicate their safety for human trials. However, berberine could interact with magnoflorine or other constituents which could further increase the tissue absorption rates. This should be noticed by other clinical trials or advanced studies to avoid adverse effects. It is also noticeable that different processing techniques or “Pao Zhi” could render the herb with different kind of therapeutic effects. For the raw PC, it could downregulate CYP1A2 and activate CYP3 A4. As for rice-wine and salt-water processed PC, they can alter the activities of cytochrome P450. Also, for rice-wine processed PC alone, it can counter the inhibitory effect of CYP1A2 and promote the induction of CYP3A4. For toxicology, there is no affirmative conclusion in terms of PC and its species' absolute clinical safety. Therefore, safety precautionary measures are still required for vulnerable groups of people. For the processed PC and its species, berberrubine, limonin, and obacunone became targeted chemical constituents for authentication of charred PC, PC, and its subspecies. Furthermore, obacunone and obaculactone are probably responsible for antiatopic dermatitis effect [56].

## 8. Conclusions

In summary, the compounds of the crude bark of PC and its species have showcased a wide range of pharmacological effects. Pharmacological efficacies of PC are supported by its diverse class of alkaloids, limonoids, phenolic acid, quinic acid, lignans, and flavonoid. Through this review, in total, 140 chemical compounds had been summarized. Among these compounds are 18 compounds from PCC, 44 compounds from PCS, 34 compounds from PAC, and 84 compounds from PAR. However, more studies are still needed to demonstrate more knowledge to allow a better understanding of this herb and its species.

## Figures and Tables

**Table 1:** Summary of chemical constituents isolated from PC and its different species (140 compounds).

Compound derivatives	Chemical compounds	Methods	References	Original species
Alkaloid	Berberine	(i) UPLC-ESI-Q-TOF-MS	(i) [8]	(i) PAC
		(ii) HPLC-DAD-ESI-MS^2^	(ii) [9]	(ii) PCS and PAR
		(iii) UPLC-Q/TOF-HDMS	(iii) [10]	(iii) PAC
		(iv) HPLC-TLC- NMR-EI-MS	(iv) [11]	(iv) PAR
		(v) HPLC-DAD-MS	(v) [12]	(v) PAR
		(vi) N/A	(vi) [13]	(vi) PAC and PCS
	8,13-dioxo-14-butoxycanadine	CC	[14]	PCS
	Berberrubine	UPLC-ESI-Q-TOF-MS	[8]	PAC
	Berberastine	UPLC-Q/TOF-HDMS	[10]	PAC
	Bis-[4-(dimethylamino)phenyl]methanone	HPLC-ESI-MS/MS	[15]	PAR
	Brucine	CE	[16]	PCS
	Δ^7^-dehydrosophoramine	N/A	[17]	PAC
	Dihydrocyclobuxine-D	HPLC-ESI-MS/MS	[15]	PAR
	3,4-dihydro-1-[(4-hydroxyphenyl)methyl]-7-methoxy-2-methyl-8-isoquinolinol	HPLC-ESI-MS/MS	[15]	PAR
	3,4-dihydro-1-[(4-hydroxyphenyl)methyl]-7-methoxy-2-methyl-6-isoquinolinol	HPLC-ESI-MS/MS	[15]	PAR
	7,8-dihydroxyrutaecarpine	HPLC-ESI-MS/MS	[15]	PAR
	4-dimethylamino-4 -isopropylbenzene	HPLC-ESI-MS/MS	[15]	PAR
	Evodiamine	HPLC-ESI-MS/MS	[15]	PAR
	Palmatine	(i) UPLC-ESI-Q-TOF-MS	(i) [8]	(i) PAC
		(ii) HPLC-DAD-ESI-MS^2^	(ii) [9]	(ii) PCS and PAR
		(iii) UPLC-Q/TOF-HDMS	(iii) [10]	(iii) PCC
		(iv) HPLC-DAD-MS	(iv) [12]	(iv) PAR
		(v) N/A	(v) [18]	(v) PCS
	Tetrahydropalmatine	UPLC-ESI-Q-TOF-MS	[8]	PAC
	Tetrahydroberberine	HPLC-ESI-MS/MS	[15]	PAR
	Phellodendrine	(i) UPLC-ESI-Q-TOF-MS	(i) [8]	(i) PAC
		(ii) HPLC-DAD-ESI-MS^2^	(ii) [9]	(ii) PCS and PAR
		(iii) UPLC-Q/TOF-HDMS	(iii) [10]	(iii) PAC
		(iv) HPLC-DAD-MS	(iv) [12]	(iv) PAR
		(v) N/A	(v) [18]	(v) PAC and PCS
	Magnocurarine	(i) HPLC-ESI-MS/MS	(i) [15]	(i) PAR
	Magnoflorine	(i) HPLC-DAD-ESI-MS^2^	(i) [9]	(i) PCS and PAR
		(ii) UPLC-Q/TOF-HDMS	(ii) [10]	(ii) PCC
		(iii) HPLC-DAD-MS	(iii) [12]	(iii) PAR
		(iv) N/A	(iv) [19]	(iv) PCS
	Jatrorrhizine or Neprotin, 2,9,10-Trimethoxy-5,6-dihydroisoquinolino[2,1-b]isoquinolin-7-ium-3-ol	(i) UPLC-ESI-Q-TOF-MS	(i) [8]	(i) PAC
		(ii) HPLC-DAD-ESI-MS^2^	(ii) [9]	(ii) PCS and PAR
		(iii) HPLC-DAD-MS	(iii) [12]	(iii) PAR
		(iv) N/A	(iv) [19]	(iv) PAC
	13-methoxyjatrorrhizine	HPLC-ESI-MS/MS	[15]	PAR
	*Ƴ*-Fagarine	CC	[20]	PAR
	Canthin-6-one	CC	[20]	PAR
	4-methoxy-N-methyl-2-quinolone	CC	[20]	PAR
	Oxypalmatine	CC	[20]	PAR
	Candicine	HPLC-DAD-ESI-MS^2^	[9]	PAR
	Lotusine	(i) HPLC-DAD-ESI-MS^2^	(i) [9]	(i) PCS and PAR
		(ii) UPLC-Q/TOF-HDMS	(ii) [10]	(ii) PAC
	N-methylhigenamine-7-O-glucopyranoside	HPLC-ESI-MS/MS	[15]	PAR
	N-methylhigenamine-7-O-*β*-D-glucopyranoside	HPLC-DAD-ESI-MS^2^	[9]	PAR
	(−)-Oblongine	(i) HPLC-DAD-ESI-MS^2^	(i) [9]	(i) PCS and PAR
		(ii) UPLC-Q/TOF-HDMS	(ii) [10]	(ii) PCC
	Isomer-of-berberine	HPLC-DAD-ESI-MS^2^	[9]	PAR
	Isomer-of-magnoflorine	HPLC-DAD-ESI-MS^2^	[9]	PCS and PAR
	Isomer-of-palmatine	HPLC-DAD-ESI-MS^2^	[9]	PAR
	Tetrahydroreticuline	HPLC-DAD-ESI-MS^2^	[9]	PAR
	Tetrahydrojatrorrhizine	HPLC-DAD-ESI-MS^2^	[9]	PCS and PAR
	Menisperine	(i) HPLC-DAD-ESI-MS^2^	(i) [9]	(i) PCS and PAR
		(ii) UPLC-Q/TOF-HDMS	(ii) [10]	(ii) PCC
		(iii) N/A	(iii) [19]	(iii) PAC
	(+) N-methylcorydine	HPLC-DAD-ESI-MS^2^	[9]	PAR
	N-methyl Tetrahydropalmatine	HPLC-ESI-MS/MS	[15]	PAR
	N-methylflindersine	HPLC-ESI-MS/MS	[15]	PAR
	Litcubine	HPLC-DAD-ESI-MS^2^	[9]	PAR
	Hydroxyl-palmatine	HPLC-DAD-ESI-MS^2^	[9]	PAR
	11-hydroxylpalmatine	HPLC-ESI-MS/MS	[15]	PAR
	13-hydroxypalmatine	HPLC-ESI-MS/MS	[15]	PAR
	7-hydroxy-8-methoxydedihydrorutaecarpine	HPLC-ESI-MS/MS	[15]	PAR
	Tetrahydropalmatine	HPLC-DAD-ESI-MS^2^	[9]	PAR
	Xanthoplanine	HPLC-DAD-ESI-MS^2^	[9]	PAR
	N-methylphoebine	HPLC-DAD-ESI-MS^2^	[9]	PAR
	Columbamine	HPLC-DAD-ESI-MS^2^	[9]	PCS and PAR
	Dihydroxyl-jatrorrhizine	HPLC-DAD-ESI-MS^2^	[9]	PAR
	Epiberberine	HPLC-DAD-ESI-MS^2^	[9]	PAR
	(6aS)-1,2,10,11-tetramethoxy-6,6-dimethyl-5,6,6a,7-tetrahydro-4H-dibenzo[de, g] quinolinium	UPLC-Q/TOF-HDMS	[10]	PCC
	Dasycarpamin	UPLC-Q/TOF-HDMS	[10]	PCC
	Pteleine	HPLC-UV	[21]	PAR
	(-)-(R)-platydesmin	HPLC-UV	[21]	PAR
	Noroxyhydrastinine	HPLC-UV	[21]	PAR
	Chilenine	HPLC-UV	[21]	PAR
	Rutecarpine	HPLC-ESI-MS/MS	[15]	PAR
	Skimmianine	HPLC-ESI-MS/MS	[15]	PAR
	Tembetarine	HPLC-ESI-MS/MS	[15]	PAR
	Tetramethyl-O-scutellarin	UPLC-Q/TOF-HDMS	[10]	PCC
	5,8,13,13a-Tetrahydro-2,9,10,11-tetrahydroxy-3-methoxy-7-methyl-6H-dibenzo[a,g]quinolizinium	HPLC-ESI-MS/MS	[15]	PAR
	*Ƴ*-hydroxybutenolide derivatives II	UPLC-Q/TOF-HDMS	[10]	PCC

Isoquinoline alkaloid	Armepavine	UPLC-ESI-Q-TOF-MS	[8]	PAC
	Demethyleneberberine	UPLC-ESI-Q-TOF-MS	[8]	PAC
	8-oxoberberine	HPLC-ESI-MS/MS	[15]	PAR
	8-oxoepiberberine	HPLC-ESI-MS/MS	[15]	PAR
	8-oxopalmatine	HPLC-ESI-MS/MS	[15]	PAR
	Oxyberberine	HPLC	[20]	PAR
	Oxypalmatine	HPLC	[20]	PAR

Limonoid	Kihadanin B	N/A	[19]	PAC
	Niloticin	N/A	[18]	PAC
	Niloticin acetate	N/A	[18]	PCS
	Obaculactone or limonin	(i) UPLC-ESI-Q-TOF-MS	(i) [8]	(i) PAC
		(ii) CC	(ii) [20]	(ii) PAR
		(iii) UPLC-Q/TOF-HDMS	(iii) [10]	(iii) PCC
		(iv) HPLC-TLC- NMR- EI-MS	(iv) [11]	(iv) PAC
		(v) N/A	(v) [18]	(v) PAC
	Derivative of obaculactone	UPLC-Q/TOF-HDMS	[10]	PCC
	Obacunone or Obacunoic acid	(i) CC	(i) [20]	(i) PAR
		(ii) UPLC-Q/TOF-HDMS	(ii) [10]	(ii) PCC
		(iii) HPLC-TLC- NMR- EI-MS	(iii) [11]	(iii) PAC
		(iv) HPLC-DAD-ESI-MS^2^	(iv) [9]	(iv) PAR
		(v) N/A	(v) [18]	(v) PAC
	12*α*-hydroxylimonin	CC	[20]	PAR
	Piscidinol A	N/A	[18]	(i) PCS
	Rutaevin	(i) HPLC-DAD-ESI-MS^2^	(i) [9]	(i) PAR
		(ii) UPLC-Q/TOF-HDMS	(ii) [10]	(ii) PCC
	Coniferin	HPLC-DAD-ESI-MS^2^	[9]	PAR
	Vanilloloside	HPLC-DAD-ESI-MS^2^	[9]	PAR
	N-methyltetrahydrocolumbamine	UPLC-Q/TOF-HDMS	[10]	PCC

N-acyl amines	Herculin	N/A	[19]	PAC

Phenolic acid	Ferulic acid	(i) HPLC-DAD-ESI-MS^2^	(i) [9]	(i) PCS and PAR
		(ii) HPLC-TLC- NMR- EI-MS	(ii) [11]	(ii) PAC
	Methyl ferulate	CC	[4]	PCS
	Protocatechuic acid	CC	[4]	PCS

Quinic acid	Quinic acid	UPLC-Q/TOF-HDMS	[10]	PAC
	Neo-chlorogenic acid	HPLC-DAD-ESI-MS^2^	[9]	PCS and PAR
	3-O-feruloylquinic acid	(i) HPLC-DAD-ESI-MS^2^	(i) [9]	(i) PCS and PAR
		(ii) UPLC-Q/TOF-HDMS	(ii) [10]	(ii) PAC
	3-O-feruloylquinic acid glucoside	UPLC-Q/TOF-HDMS	[10]	PCC
	4-O-feruloylquinic acid	HPLC	[22]	PCS
	5-O-feruloylquinic acid	HPLC	[22]	PCS
	Chlorogenic acid	(i) HPLC-DAD-ESI-MS^2^	(i) [9]	(i) PCS and PAR
		(ii) UPLC-Q/TOF-HDMS	(ii) [10]	(ii) PAC
		(iii) HPLC	(iii) [23]	(iii) PAR
		(iv) HPLC-DAD-MS	(iv) [12]	(iv) PAR
	Methyl 3-*O*-feruloylquinate	HPLC-DAD-ESI-MS^2^	[9]	PAR
	Methyl 5-O-feruloylquinate	NMR	[24]	PAR
	3-Feruoyl-4-caffeoylquinic acid	HPLC-DAD-ESI-MS^2^	[9]	PAR
	Sanleng acid	NMR	[25]	PAC

Hydroxycinnamic acid	Caffeic Acid Methyl Ester	HPLC	[22]	PCS

Phytosterol	*β*-Sitosterol	N/A	[18]	PAC

Lignan	(+/-)-lyoniresinol	HPLC-DAD-ESI-MS^2^	[9]	PAR
	(+/-)-5,5′-dimethoxylariciresinol-4-*O*-glucoside	(i) HPLC-DAD-ESI-MS^2^	(i) [9]	(i) PCS and PAR
		(ii) UPLC-Q/TOF-HDMS	(ii) [10]	(ii) PCC
	Syringaresinol di-O-*β*-D-glucopyranoside	HPLC-DAD-ESI-MS^2^	[9]	PCS and PAR
	(-)-Syringaresinol	CC	[4]	PCS

Flavonoid	Amurensin	N/A	[13]	PAC
	Quercetin	HPLC-TLC- NMR- EI-MS	[11]	PAC
	Phellamurin	N/A	[18]	PAC
	Phellatin	N/A	[18]	PAC
	Phellavin	N/A	[18]	PAC
	Phellodendroside	N/A	[18]	PAC
	*β*-anhydronoricaritin	UV	[26]	PAR
	Icariside-1	UV	[26]	PAR
	Phellamuretin	UV	[26]	PAR
	Phelloside	UV	[26]	PAR
	Dihydrophelloside	UV	[26]	PAR
	Isovaleric acid	UV	[26]	PAR
	Kaempferol	UV	[26]	PAR
	D-glucose	UV	[26]	PAR

Rutaceae	Noricariside	N/A	[18]	PAC

Stigmastane	7-Dehydrostigmasterol	N/A	[17]	PAC

Coumarin	3-acetyl-3,4-dihydro-5,6-dimethoxy-1H-2-benzopyran-1-one	CC	[27]	PCS

Monosaccharide	Syringin	NMR	[25]	PAC
	Daucosterol	NMR	[25]	PAC

Paraben	N-propyl paraben	HPLC	[5]	PC

Phenolic lactone	Phellolactone	CC	[4]	PCS

Ferulate	N-butyl Ferulate	CC	[4]	PCS
	Amurenlactone A	CC	[4]	PCS
	Amurenamide A	NMR	[25]	PAC

Hydroxybenzaldehyde	4-hydroxybenzaldehyde	CC	[4]	PCS

Phenolic aldehyde	Vanillin	CC	[4]	PCS

Glycoside	Sinapyl aldehyde-4-O-beta-D-glucopyranoside	NMR	[24]	PAR
	3,4,5-Trimetoxyphenol O-*β*-D-glucopyranoside	HPLC	[22]	PCS
	2-methoxy-4-(2-propenyl)phenyl-beta-D-glucopyranoside	HPLC	[22]	PCS
	2-(p-hydroxyphenyl)-ethanol-1-O-*β*-D-apiofuranosyl (1–6)-O-*β*-D-glucopyranoside	HPLC-DAD-ESI-MS^2^	[9]	PCS and PAR
	2-(p-hydroxyphenyl) -ethanol-1-O-*β*-D-glucoside	UPLC-Q/TOF-HDMS	[10]	PCC
	3, 5-dihydroxybenzoicacid-O-xylopyranosyl-glucopyranoside	HPLC-DAD-ESI-MS^2^	[9]	PCS and PAR

Phenolic glycoside	Tachioside	HPLC	[22]	PCS

Glucoside	Salidroside	HPLC	[22]	PCS
	4-hydroxybenzyl alcohol	HPLC	[22]	PCS

Phenol	Methyl syringate	HPLC	[22]	PCS

Dehydrovomifoliol	(6S)-dehydrovomifoliol	HPLC	[22]	PCS
	(6R,7aR)-epiloliolide	HPLC	[22]	PCS

PAC: phellodendri amurensis cortex

PCS: phellodendron chinense schneid

PAR: phellodendron amurense rupr.

PCC: phellodendri Chinensis cortex

UPLC-ESI-Q-TOF-MS: ultra-performance liquid chromatography coupled with electrospray ionization/quadrupole-time-of-flight mass spectrometry

CC: column chromatography

CE: capillary electrophoresis

HPLC/MSD: high-performance liquid chromatography coupled with mass spectrometric detection

UPLC–Qthe -TOF-MS: ultra-performance liquid chromatography with quadrupole TOF-MS

HPLC– ESI-MS2: high-performance liquid chromatography with electrospray ionization mass spectrometry coupled with photodiode array detection

UPLC-Q/TOF-HDMS: ultra-performance liquid chromatography-quadrupole/time-of-flight high-definition mass spectrometry

UV: UV detection

EI-MS: Electron ionization mass spectrometry

NMR: Nuclear magnetic resonance

TLC: Thin-layer chromatography

HPLC-DAD-MS: high-performance liquid chromatography coupled with diode array detection and mass spectrometry

**Table 2:** Molecular formula, molecular weight and chemical structures of compounds derived from PCC species (18 compounds).

Compound derivatives	Compound	Molecular formula	Molecular weight	Chemical structures
Alkaloid	Palmatine	C_21_H_22_NO_4_^+^	352.41 g/mol	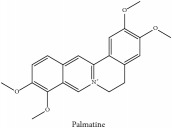
	Magnoflorine	C_20_H_24_NO_4_^+^	342.41 g/mol	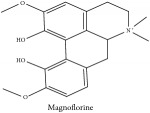
	(−)-Oblongine	C_19_H_24_NO_3_^+^	314.40 g/mol	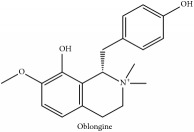
	Menisperine	C_21_H_26_NO_4_^+^	356.44 g/mol	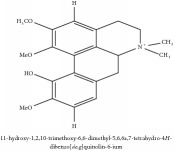
	(6aS)-1,2,10,11-tetramethoxy-6,6-dimethyl-5,6,6a,7-tetrahydro-4H-dibenzo[de, g] quinolinium	C_22_H_28_NO_4_	370.47 g/mol	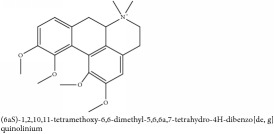
	Dasycarpamin	C_17_H_21_NO_4_	303.36 g/mol	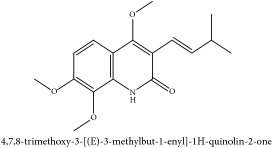
	Tetramethyl-O-scutellarin	C_19_H_18_O_6_	342.35 g/mol	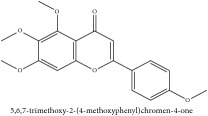

	*Ƴ*-hydroxybutenolide derivatives II	N/A		

Limonoid	Niloticin acetate	C_32_H_50_O_4_	498.75 g/mol	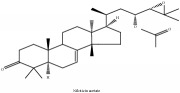
	Obaculactone or limonin	C_26_H_30_O_8_	470.52 g/mol	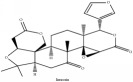
	Derivative of obaculactone	N/A		
	Obacunone or Obacunoic acid	C_26_H_30_O_7_	454.519 g/mol	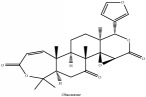
	Piscidinol A	C_30_H_50_O_4_	474.73 g/mol	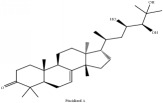
	Rutaevin	C_26_H_30_O_9_	486.52 g/mol	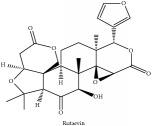
	N–methyltetrahydrocolumbamine	C_21_H_26_NO_4_^+^	356.44 g/mol	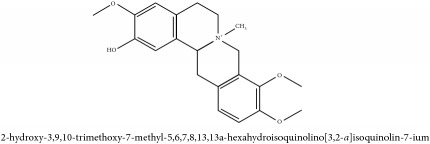

Phenolic acid	2-(p-hydroxyphenyl) -ethanol-1-O-*β*-D-glucoside	C_9_H_12_O	136.19 g/mol	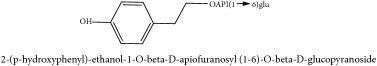

Quinic acid	3-O-feruloylquinic acid glucoside	C_22_H_28_O_15_	532.45 g/mol	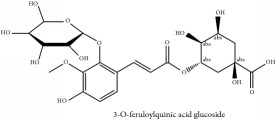

Lignan	(+/-)-5,5′-dimethoxylariciresinol-4-*O*-glucoside	C_28_H_38_O_13_	582.6 g/mol	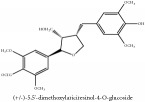

**Table 3:** Molecular formula, molecular weight and chemical structures of compounds derived from PCS species (44 compounds).

Compound derivatives	Compound	Molecular formula	Molecular weight	Chemical structures
Alkaloid	Berberine	C_20_H_18_NO_4_^+^	336.37 g/mol	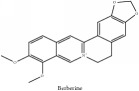
	8,13-dioxo-14-butoxycanadine	C_24_H_25_NO_7_	439.46 g/mol	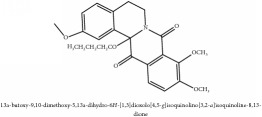
	Brucine	C_23_H_26_N_2_O_4_	394.47 g/mol	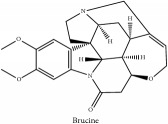
	Palmatine	C_21_H_22_NO_4_^+^	352.41 g/mol	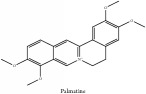
	Phellodendrine	C_20_H_24_NO_4_^+^	342.41 g/mol	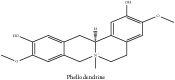
	Magnoflorine	C_20_H_24_NO_4_^+^	342.41 g/mol	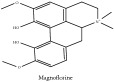
	Jatrorrhizine or Neprotin, 2,9,10-Trimethoxy-5,6-dihydroisoquinolino[2,1-b]isoquinolin-7-ium-3-ol	C_20_H_19_NO_4_	337.38 g/mol	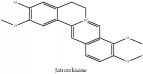
	Lotusine	C_19_H_24_NO_3_^+^	314.40 g/mol	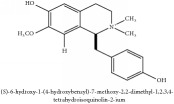
	(−)-Oblongine	C_19_H_24_NO_3_^+^	314.40 g/mol	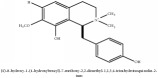
	Isomer-of-magnoflorine	N/A		
	Tetrahydrojatrorrhizine	C_20_H_23_NO_4_	341.41 g/mol	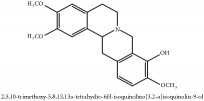
	Menisperine	C_21_H_26_NO_4_^+^	356.44 g/mol	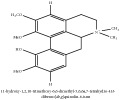
	Columbamine	C_20_H_20_NO_4_^+^	338.38 g/mol	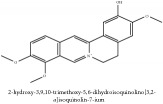
	Niloticin acetate	C_32_H_50_O_4_	498.75 g/mol	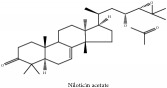
	Piscidinol A	C_30_H_50_O_4_	474.73 g/mol	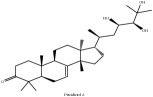

Quinic acid	Chlorogenic acid	C_16_H_18_O_9_	354.31 g/mol	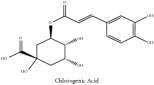
	Neo-chlorogenic acid	C_16_H_18_O_9_	354.31 g/mol	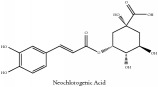
	3-O-feruloylquinic acid	C_17_H_20_O_9_	368.34 g/mol	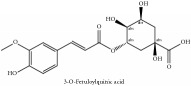
	4-O-feruloylquinic acid	C_17_H_20_O_9_	368.34 g/mol	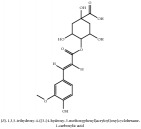
	5-O-feruloylquinic acid	C_17_H_20_O_9_	368.34 g/mol	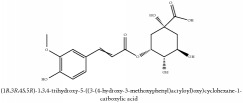

Hydroxycinnamic acid	Caffeic Acid Methyl Ester	C_10_H_10_O_4_	194.19 g/mol	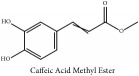

Phenolic acid	Ferulic acid	C_10_H_10_O_4_	194.19 g/mol	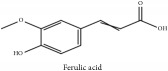
	Methyl ferulate	C_11_H_12_O_4_	208.21 g/mol	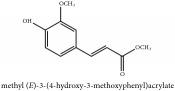
	Protocatechuic acid	C_7_H_6_O_4_	154.12 g/mol	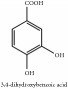

Lignan	(+/-)-5,5′-dimethoxylariciresinol-4-*O*-glucoside	C_28_H_38_O_13_	582.6 g/mol	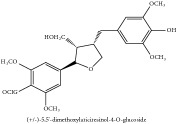
	Syringaresinol di-O-*β*-D-glucopyranoside	C_33_H_44_O_18_	728.70 g/mol	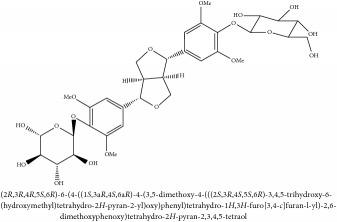
	(-)-syringaresinol	C_22_H_26_O_8_	418.44 g/mol	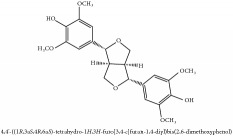

Coumarin	3-acetyl-3,4-dihydro-5,6-dimethoxy-1H-2-benzopyran-1-one	C_13_H_14_O_5_	250.25 g/mol	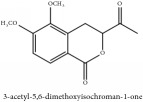

Paraben	N-propyl paraben	C_10_H_12_O_3_	180.20 g/mol	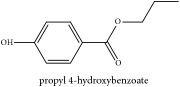

Phenolic lactone	Phellolactone	C_13_H_14_O_8_	298.25 g/mol	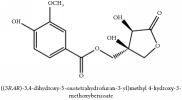

Ferulate	N-butyl Ferulate	C_14_H_18_O_4_	250.29 g/mol	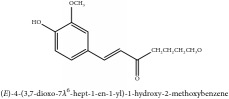
	Amurenlactone A	C_17_H_20_O_9_	368.34 g/mol	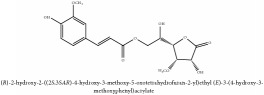

Hydroxybenzaldehyde	4-hydroxybenzaldehyde	C_7_H_8_O_2_	124.14 g/mol	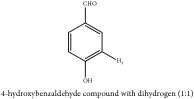

Phenolic aldehyde	Vanillin	C_8_H_8_O_3_	152.15 g/mol	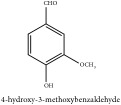

Glycoside	3,4,5-trimetoxyphenol O-*β*-D-glucopyranoside	C_9_H_11_O_3_	167.18 g/mol	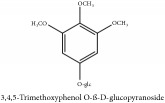
	2-methoxy-4-(2-propenyl)phenyl-beta-D-glucopyranoside	C_16_H_22_O_7_	326.35 g/mol	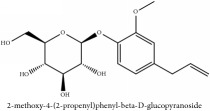
	2-(p-hydroxyphenyl)-ethanol-1-O-*β*-D-apiofuranosyl (1–6)-O-*β*-D-glucopyranoside	C_9_H_12_O	136.19 g/mol	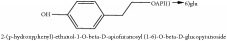
	3, 5-dihydroxybenzoicacid-O-xylopyranosyl-glucopyranoside	C_8_H_8_O_3_	152.15 g/mol	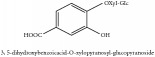

Phenolic glycoside	Tachioside	C_12_H_16_O_8_	288.25 g/mol	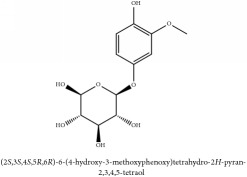

Glucoside	Salidroside	C_14_H_20_O_7_	300.31 g/mol	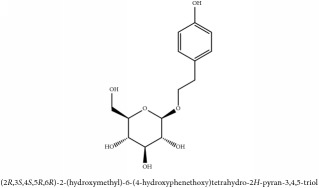
	4-Hydroxybenzyl alcohol	C_7_H_8_O_2_	124.14 g/mol	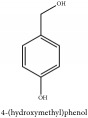

Phenol	Methyl Syringate	C_10_H_12_O_5_	212.20 g/mol	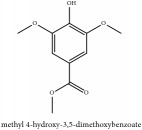

Dehydrovomifoliol	(6S)-dehydrovomifoliol	C_13_H_18_O_3_	222.28 g/mol	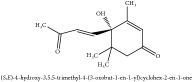
	(6R,7aR)-epiloliolide	C_11_H_16_O_3_	196.25 g/mol	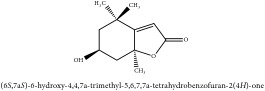

**Table 4:** Molecular formula, molecular weight and chemical structures of compounds derived from PAC species (34 compounds).

Compound derivatives	Compound	Molecular formula	Molecular weight	Chemical structures
Alkaloid	Berberine	C_20_H_18_NO_4_^+^	336.37 g/mol	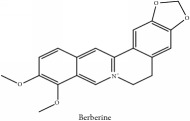
	Berberrubine	C_19_H_16_NO_4_^+^	322.34 g/mol	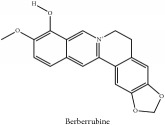
	Berberastine	C_20_H_18_NO_5_^+^	352.37 g/mol	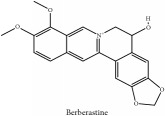
	Δ^7^-Dehydrosophoramine	C_15_H_18_N_2_O	242.32 g/mol	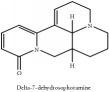
	Palmatine	C_21_H_22_NO_4_^+^	352.41 g/mol	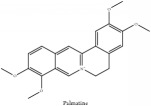
	Tetrahydropalmatine	C_21_H_25_NO_4_	355.43 g/mol	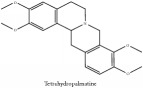
	Phellodendrine	C_20_H_24_NO_4_^+^	342.41 g/mol	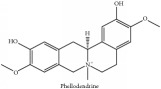
	Jatrorrhizine or Neprotin, 2,9,10-Trimethoxy-5,6-dihydroisoquinolino[2,1-b]isoquinolin-7-ium-3-ol	C_20_H_19_NO_4_	337.38 g/mol	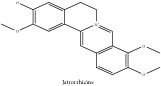
	Lotusine	C_19_H_24_NO_3_^+^	314.40 g/mol	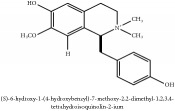
	Menisperine	C_21_H_26_NO_4_^+^	356.44 g/mol	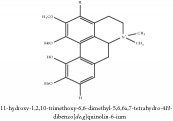

Isoquinoline alkaloid	Armepavine	C_19_H_23_NO_3_	313.40 g/mol	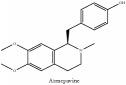
	Demethyleneberberine	C_19_H_18_NO_4_^+^	324.36 g/mol	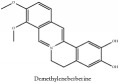

Limonoid	Kihadanin B	C_26_H_30_O_9_	486.52 g/mol	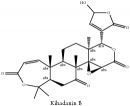
	Niloticin	C_30_H_48_O_3_	456.71 g/mol	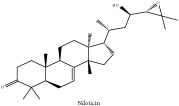
	Obaculactone or limonin	C_26_H_30_O_8_	470.52 g/mol	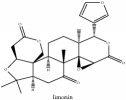
	Obacunone or Obacunoic acid	C_26_H_30_O_7_	454.52 g/mol	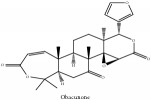

N-acyl amines	Herculin	C_16_H_29_NO	251.41 g/mol	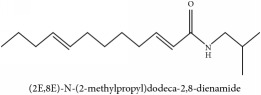

Phenolic acid	Ferulic acid	C_10_H_10_O_4_	194.19 g/mol	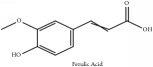

Quinic acid	Quinic acid	C_7_H_12_O_6_	192.17 g/mol	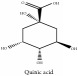
	3-O-feruloyl quinic acid or 5-O-feruloyl quinic acid	C_17_H_20_O_9_	368.34 g/mol	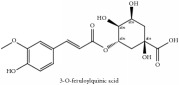
	Chlorogenic acid	C_16_H_18_O_9_	354.31 g/mol	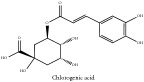
	Sanleng acid	C_18_H_34_O_5_	330.47 g/mol	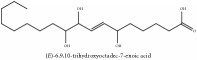

Phytosterol	*β*-sitosterol	C_29_H_50_O	414.72 g/mol	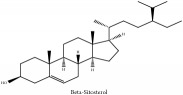

Flavonoid	Amurensin	C_26_H_30_O_12_	534.51 g/mol	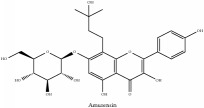
	Quercetin	C_15_H_10_O_7_	302.24 g/mol	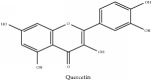
	Phellamurin	C_26_H_30_O_11_	518.52 g/mol	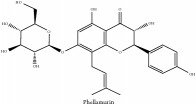
	Phellatin	C_26_H_30_O_12_	534.51 g/mol	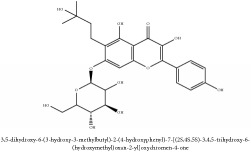
	Phellavin	C_26_H_32_O_12_	536.53 g/mol	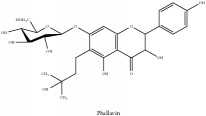
	7-dehydrostigmasterol	C_29_H_50_O	414.72 g/mol	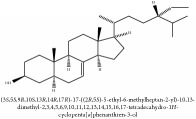

Rutaceae	Noricariside	C_26_H_30_O_12_	534.51 g/mol	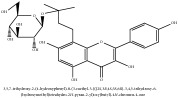

Stigmastane	Phellodendroside	C_26_H_30_O_11_	518.52 g/mol	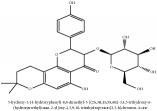

Monosaccharide	Syringin	C_17_H_24_O_9_	372.37 g/mol	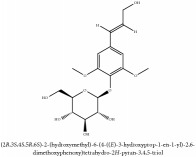
	Daucosterol	C_34_H_58_O_6_	562.83 g/mol	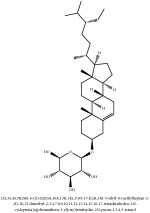

Ferulate	Amurenamide A	C_17_H_25_NO_9_	387.39 g/mol	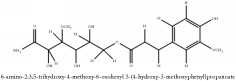

**Table 5:** Molecular formula, molecular weight and chemical structures of compounds derived from PAR species (84 compounds).

Compound derivatives	Compound	Molecular formula	Molecular weight	Chemical structures
Alkaloid	Berberine	C_20_H_18_NO_4_^+^	336.37 g/mol	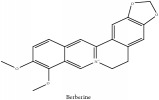
	Bis-[4-(dimethylamino)phenyl]methanone	C_17_H_20_N_2_O	268.36 g/mol	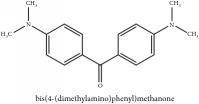
	Dihydrocyclobuxine-D	C_25_H_44_N_2_O	388.64 g/mol	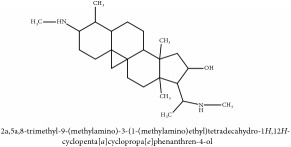
	3,4-Dihydro-1-[(4-hydroxyphenyl)methyl]-7-methoxy-2-methyl-8-isoquinolinol	C_20_H_24_NO_4_^+^	342.41 g/mol	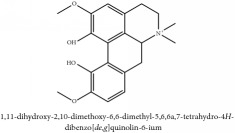
	3,4-Dihydro-1-[(4-hydroxyphenyl)methyl]-7-methoxy-2-methyl-6-isoquinolinol	C_20_H_17_NO_5_	351.36 g/mol	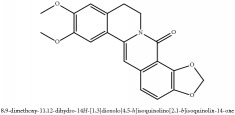
	7,8-Dihydroxyrutaecarpine	C_18_H_13_N_3_O_3_	319.32 g/mol	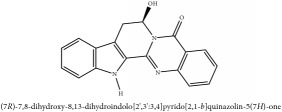
	4-Dimethylamino-4 -isopropylbenzene	C_18_H_22_NO^+^	268.38 g/mol	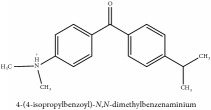
	Evodiamine	C_19_H_17_N_3_O	303.37 g/mol	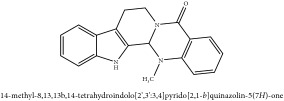
	Palmatine	C_21_H_22_NO_4_^+^	352.41 g/mol	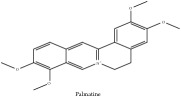
	Pteleine	C_13_H_13_NO_3_	231.25 g/mol	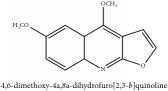
	(-)-(R)-platydesmin	C_15_H_19_NO_3_	261.32 g/mol	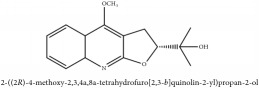
	Noroxyhydrastinine	C_10_H_9_NO_3_	191.19 g/mol	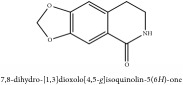
	Chilenine	C_19_H_15_NO_7_	369.33 g/mol	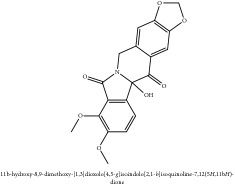
	Phellodendrine	C_20_H_24_NO_4_^+^	342.41 g/mol	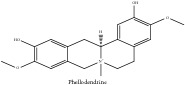
	Magnocurarine	C_19_H_24_NO_3_^+^	314.40 g/mol	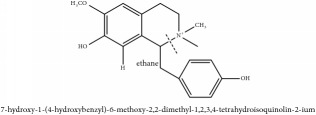
	Magnoflorine	C_20_H_24_NO_4_^+^	342.41 g/mol	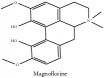
	Jatrorrhizine or Neprotin, 2,9,10-Trimethoxy-5,6-dihydroisoquinolino[2,1-b]isoquinolin-7-ium-3-ol	C_20_H_19_NO_4_^+^	337.38 g/mol	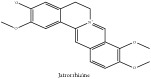
	13-Methoxyjatrorrhizine	C_21_H_22_NO_5_^+^	368.41 g/mol	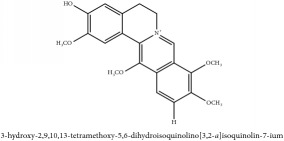
	*Ƴ*-Fagarine	C_13_H_11_NO_3_	229.24 g/mol	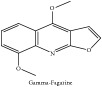
	Canthin-6-one	C_14_H_8_N_2_O	220.23 g/mol	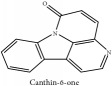
	4-Methoxy-N-methyl-2-quinolone	C_11_H_11_NO_2_	189.21 g/mol	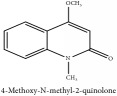
	Candicine	C_10_H_16_NO^+^	166.24 g/mol	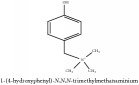
	Lotusine	C_19_H_24_NO_3_^+^	314.40 g/mol	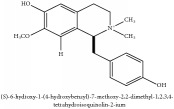
	N-Methylhigenamine-7-O-glucopyranoside	C_17_H_20_N_2_O	268.36 g/mol	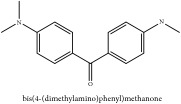
	N-methylhigenamine-7-O-*β*-D-glucopyranoside	N/A	N/A	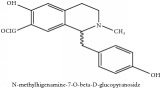
	(−)-Oblongine	C_19_H_24_NO_3_^+^	314.40 g/mol	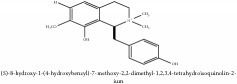
	Isomer-of-berberine	N/A		
	Isomer-of-magnoflorine			
	Isomer-of-palmatine			
	Tetrahydroreticuline	C_19_H_22_NO_4_^+^	328.39 g/mol	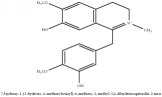
	Tetrahydrojatrorrhizine	C_20_H_23_NO_4_	341.41 g/mol	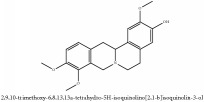
	Menisperine	C_21_H_26_NO_4_^+^	356.44 g/mol	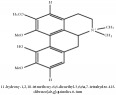
	(+) N-methylcorydine	C_21_H_26_NO_4_^+^	356.44 g/mol	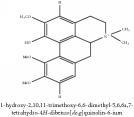
	N-Methyl Tetrahydropalmatine	C_22_H_28_NO_4_^+^	370.47 g/mol	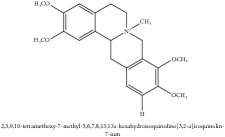
	N-Methylflindersine	C_15_H_15_NO_2_	241.29 g/mol	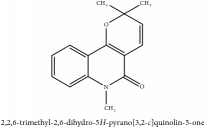
	Litcubine	C_19_H_22_NO_4_^+^	328.39 g/mol	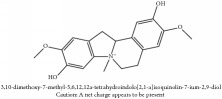
	Hydroxyl-palmatine	C_22_H_22_O_5_	366.41 g/mol	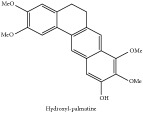
	11-Hydroxylpalmatine	C_21_H_22_NO_5_^+^	368.41 g/mol	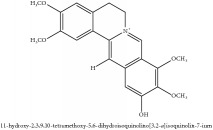
	13-Hydroxypalmatine	C_21_H_22_NO_5_^+^	368.41 g/mol	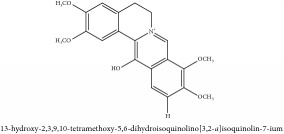
	7-Hydroxy-8-methoxydedihydrorutaecarpine	N/A		
	Tetrahydropalmatine	C_21_H_25_NO_4_	355.43 g/mol	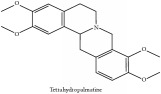
	5,8,13,13a-Tetrahydro-2,9,10,11-tetrahydroxy-3-methoxy-7-methyl-6H-dibenzo[a,g]quinolizinium	C_21_H_20_NO_6_^+^	382.39 g/mol	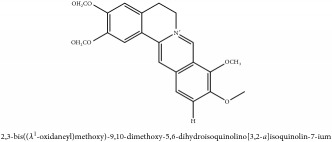
	Tetrahydroberberine	C_20_H_21_NO_4_	339.39 g/mol	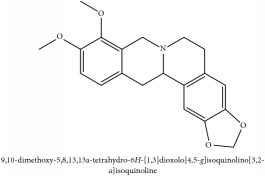
	Xanthoplanine	C_21_H_26_NO_4_^+^	356.44 g/mol	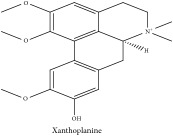
	N-methylphoebine	C_22_H_26_NO_5_^+^	384.45 g/mol	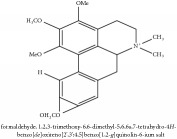
	Columbamine	C_20_H_20_NO_4_^+^	338.38 g/mol	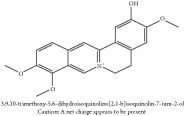
	Dihydroxyl-jatrorrhizine	N/A		
	Epiberberine	C_20_H_18_NO_4_^+^	336.37 g/mol	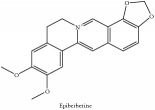
	Rutecarpine	C_18_H_13_N_3_O	287.32 g/mol	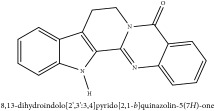
	Skimmianine	C_14_H_14_NO_4_^+^	260.27 g/mol	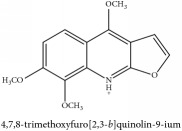
	Tembetarine	C_21_H_22_NO_4_^+^	352.41 g/mol	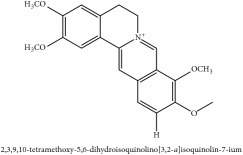
	8-Oxoberberine	C_20_H_17_NO_5_	351.36 g/mol	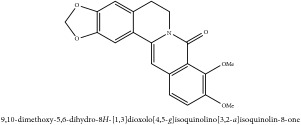
	8-Oxoepiberberine	C_20_H_17_NO_5_	351.36 g/mol	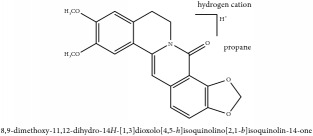
	8-Oxopalmatine	C_21_H_21_NO_5_	367.40 g/mol	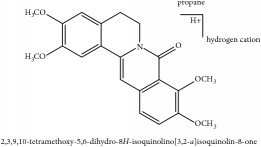
	Oxyberberine	C_20_H_17_NO_5_	351.36 g/mol	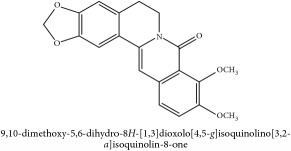
	Oxypalmatine	C_21_H_21_NO_5_	367.40 g/mol	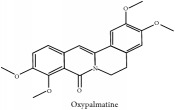

Limonoid	Obaculactone or limonin	C_26_H_30_O_8_	470.52 g/mol	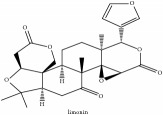
	Obacunone or Obacunoic acid	C_26_H_30_O_7_	454.52 g/mol	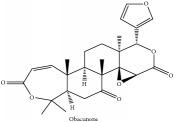
	12*α*-hydroxylimonin	C_26_H_30_O_9_	470.52 g/mol	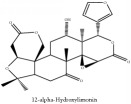
	Rutaevin	C_26_H_30_O_9_	486.52 g/mol	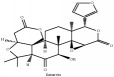
	Coniferin	C_16_H_22_O_8_	342.34 g/mol	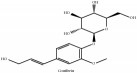
	Vanilloloside	C_14_H_20_O_8_	316.31 g/mol	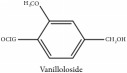

Phenolic acid	2-(p-hydroxyphenyl)-ethanol-1-O-*β*-D-apiofuranosyl (1–6)-O-*β*-D-glucopyranoside	C_9_H_12_O	136.19 g/mol	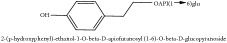
	3, 5-dihydroxybenzoicacid-O-xylopyranosyl-glucopyranoside	C_8_H_8_O_3_	152.15 g/mol	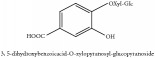
	Ferulic acid	C_10_H_10_O_4_	194.19 g/mol	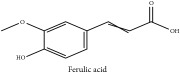

Quinic acid	Neochlorogenic acid	C_16_H_18_O_9_	354.31 g/mol	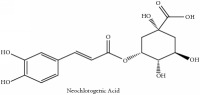
	3-O-feruloyl quinic acid or 5-O-feruloyl quinic acid	C_17_H_20_O_9_	368.34 g/mol	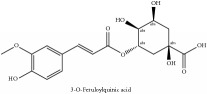
	Chlorogenic acid	C_16_H_18_O_9_	354.31 g/mol	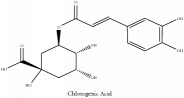
	Methyl 3-*O*-feruloylquinate	C_18_H_22_O_9_	382.37 g/mol	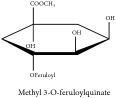
	Methyl 5-O-feruloylquinate	C_8_H_13_O_6_	205.19 g/mol	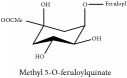
	3-Feruoyl-4-caffeoylquinic acid	C_26_H_26_O_12_	530.48 g/mol	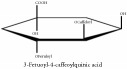

Lignan	(+/-)-lyoniresinol	C_22_H_28_O_8_	420.46 g/mol	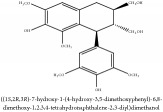
	(+/-)-5,5′- dimethoxylariciresinol-4-*O*-glucoside	C_28_H_38_O_13_	582.6 g/mol	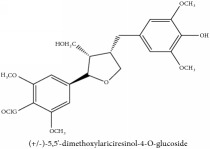
	Syringaresinol di-O-*β*-D-glucopyranoside	C_33_H_44_O_18_	728.70 g/mol	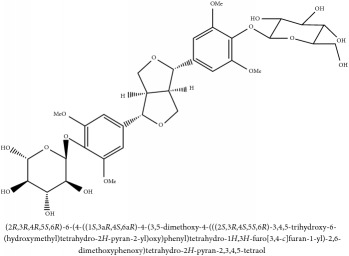

Flavonoid	*β*-anhydronoricaritin	N/A		
	Icariside-1	C_26_H_28_O_11_	516.50 g/mol	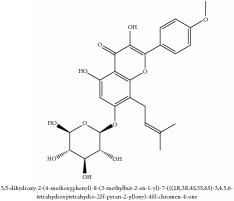
	Phellamuretin	C_20_H_20_O_6_	356.37 g/mol	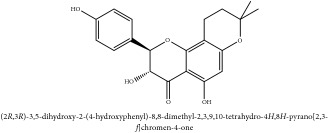
	Phelloside	C_31_H_40_CrO_17_	736.64 g/mol	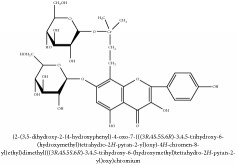
	Dihydrophelloside	C_31_H_44_CrO_17_	740.67 g/mol	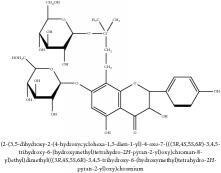
	Isovaleric acid	C_5_H_10_O_2_	102.13 g/mol	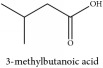
	Kaempferol	C_15_H_10_O_6_	286.24 g/mol	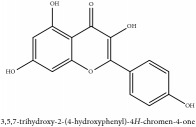
	D-glucose	C_6_H_12_O_6_	180.16 g/mol	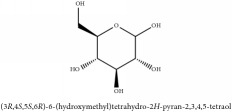

Paraben	N-propyl paraben	C_10_H_12_O_3_	180.20 g/mol	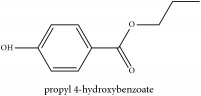

Glycoside	Sinapyl aldehyde-4-O-beta-D-glucopyranoside	C_13_H_14_O_6_	266.25 g/mol	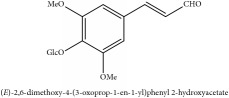

**Table 6:** Pharmacological activities of PC and processed PC.

Pharmacological Activity	Tested Substance	*In vivo/ In vitro*	Model or sample	Active concentration	Administration (In vivo)	References
Anti-inflammatory effect	PAR extract with voucher specimens	*In vitro*	BV2 cells (Mouse microglial cell line)	100 *μ*g/ml		[28]

	PCC extract	*In vivo, in vitro*	12-*O*-Tetradecanoylphorbol-13-acetate-induced mouse ear edema	(i) TPA and AA tests: 0.5 mg/ear (ii) TPA multiple application: 1 mg/ear (iii) DTH test: 1 mg/ear	Topical application	[29]

	PCC extract with voucher specimens	*In vivo, in vitro*	lipopolysaccharides-induced systemic inflammation mice model and macrophage RAW 264.7 cells	1,10,100 *μ*g/mL	Oral administration	[30]

	Ethanol extract of PCC with voucher specimens	*In vivo*	12-O-tetradecanoyl-phorbol-13-acetate- induced ear edema in mice	200-400 mg/kg	Administered intragastrically	[3]

	PAR methanol extract with voucher specimens	*In vitro, in vivo*	ICR mice and male Wistar rats	IC_50_: Methanol extract 20.9 ± 3.8 *μ*g/mL; Non-alkaloids 22.0 *μ*g/mL; Limonin 15.8 ± 5.2 *μ*m; Obakunone 2.6 ± 1.1 *μ*m	Oral administration	[11]

	PCC methanol extract with voucher specimens	*In vivo*	Lipopolysaccharides- induced acute airway inflammation on a mouse model	100, 200 and 400 mg/kg	Administrated by gavage	[31]

	Demethyleneberberine	*In vivo*	Acute colitis mice model	150,300 mg/kg	Oral administration	[32]

Anti-bacterial effect	Ethanol Extract of PAR; Aqueous extract of PAR	*In vitro*	Enterococcus faecium, Staphylococcus aureus, Streptococcus pyogenes, Escherichia coli, Klebsiella pneumonia, Pseudomonas aeruginosa	MIC and MBC: 3.676 mg/ml and 7.353 mg/ml for ethanol extract; 6.25 mg/ml and 50 mg/ml for aqueous extract		[33]

	PCC extract with voucher specimens	*In vitro*	Streptococcus. mitis, Streptococcus. sanguis, Streptococcus. mutans, Streptococcus. gingivalis	2.5 g/ml		[34]

	PCC extract	*In vitro*	Mycoplasma hominis	0.24-250 mg/ml		[35]

	Aqueous extract of PCC	*In vivo, In vitro*	*S. *Typhimurium 21 infected mouse model	2.5 or 5 mg/day	Administrated by gavage	[36]

	Berberine in PAR	*In vitro*	Staphylococcus aureus	32 to 128 *μ*g/mL		[37]

	Berberine in PCS	*In vitro*	P. aeruginosa	100 ml		[38]

	PCC	*In vitro*	Propionibacterium acnes	MIC50%: 24 *μ*g/mL MIC90%: 190 *μ*g/mL		[39]

Anti-fungal effect	Berberine hydrochloride, palmatine hydrochloride	*In vitro, In vivo*	Microsporum Canis –induced dermatitis in rabbits	MIC_s_ 1 mg/ml	Administered through the sterile pipette tip	[40]

Anti-viral effect	Ethanol extract of PAR; Aqueous extract of PAR	*In vitro*	African green monkey kidney cells	6.73 ± 0.87 mg/ml for aqueous extract; 4.26 ± 0.59 mg/ml for ethanol extract		[33]

	Aqueous extract of PAR with voucher specimens	*In vitro, in vivo*	H1N1, H5N2, H7N3 or H9N2 infected BALB/c mice	0.8 *μ*g/g in a total volume of 200 *μ*l at 1, 3 and 5 days before infection	Oral administration	[41]

Anti-tumor effect	PCS extract with voucher specimens	*In vitro*	(i) Leukemic cell lines K562 (ii) Leukemic cell lines HEL (iii) Breast cancer cell line MDA (iv) Prostate cancer cell line PC3	(i) IC_50_ of Compound 1: 7.66 ±2.08; (ii) IC_50_ of compound 3: 14.30 ± 1.93; (iii) IC_50_ of compound 4: 11.81 ± 2.79		[42]

	PCC extract	*In vivo, in vitro*	Prostate cancer cell infested Male BALB/c-nude mice model	1.6 g/kg per day for 28 days	Administered intragastrically	[43]

	Aqueous extract of PCS with voucher specimens	*In vitro In vivo*	Sarcoma 180 ascites cells implanted Mice model	2 mg/100g; 5 mg/100g; 10 mg/100g	Injected intraperitoneally daily for 10 days	[44]

Anti-gout effect	Compounds of Si-Miao-Wan (containing PCC)	*In vivo*	Male Sprague-Dawley rats	1.0 ml/100 g	Oral administration	[45]

	PCS extract with voucher specimens	*In vivo*	Uricase inhibitor potassium oxonate induced male ICR mice model	480 mg/kg	Oral or intraperitoneal administration	[46]

Anti-ulcer effect	Ethanol extract of PCC with voucher specimens	*In vivo In vitro*	Acetic acid-induced chronic gastric ulcers on Sprague Dawley rats model	30 mg/kg/day	Administered intragastrically once a day for seven days.	[47]

	Aqueous extract of PCC	*In vivo*	Ethanol-induced gastric lesions on Male Wistar rats model	100 mg/kg	Oral administration	[48]

Anti-oxidant effect	Ethanol extract of PAR; Aqueous extract of PAR	*In vitro*	For anti-microbial Enterococcus faecium, Staphylococcus aureus, Streptococcus pyogenes, Escherichia coli, Klebsiella pneumonia, and Pseudomonas aeruginosa; Anti-herpes simplex virus tested on African green monkey kidney cells	25 mg/ml		[33]

	Phellodendrine isolated from PCC extract	*In vivo*	AAPH-induced oxidative stress on zebrafish embryo model	200 *μ*g/mL	Waterborne exposure	[49]

Sun screening effect	PCC extract + 50% ethanol	*In vitro*	PC extract+50% ethanol	0.5 mg/ml		[50]

	PCC extract	*In vivo*	UVB lamp inflicted skin lesions on the dorsal of the rats model	200, 400 or 800 mg/kg, once daily for 11 days	Oral administration	[51]

Bone-growth effect	PCC extract	*In vivo*	72 intact 21-day-old female Sprague–Dawley rats	100 and 300 mg/kg	Oral administration	[52]

Hemostatic effect	PCC Carbonisatus-carbon dots	*In vivo In vitro*	Mouse tail amputation and liver scratch on male Kunming mice models	5, 2 and 1 mg*/*kg	Subcutaneous administration	[53]

Neuroprotective effect	PCC extract with voucher specimens	*In vitro*	PC-12 cells	10 and 30 *μ*g/mL		[54]
	PCC and PAC extract with voucher specimens	*In vitro*	PC-12 cells	0.1 and 1 g/ml for 2 hours		[55]

Counter-atopic dermatitis effect	Salt processed PAC extract with voucher specimens	*In vivo*	2,4-dinitrochlorobenzene induced skin lesions on the NC/Nga mice model	200 *μ*l	Topical administration	[56]

Counter-diabetic nephropathy effect	PAR aqueous extract	*In vivo*	Streptozotocin-induced diabetes on male Sprague-Dawley rats model	379 mg/kg	Oral administration	[57]

	Berberine	*In vivo*	Streptozotocin-induced diabetes on male Wistar rats model	200 mg/kg once a day for 12 weeks	Oral intubation	[58]

Immunity suppressing effect	Phellodendrine and cyclophosphamide isolated from PCC in saline water	*In vivo*	ddY mice, BALB/c mice, and Hartlay guinea pigs	(i) 0.1 ml/10 g for mice (ii) 1 ml for guinea pigs	Administered intraperitoneally	[59]

Anti-asthmatic effect	n-butyl alcohol extract of PCS with voucher specimens	*In vivo*	BALB/c mice asthmatic model induced by saline solution	The IC50 of n-butyl alcohol extract of PCS was 12.2 ± 1.3 *μ*g/mL	intranasally	[6]
